# In Situ Synthesis of Horseradish Peroxidase Nanoflower@Carbon Nanotube Hybrid Nanobiocatalysts with Greatly Enhanced Catalytic Activity

**DOI:** 10.1021/acs.langmuir.3c00260

**Published:** 2023-03-21

**Authors:** Seyma Dadi, Nimet Temur, O. Tolga Gul, Vedat Yilmaz, Ismail Ocsoy

**Affiliations:** † Department of Analytical Chemistry, Faculty of Pharmacy, Erciyes University, Kayseri 38039, Turkey; ‡ Department of Nanotechnology Engineering, Abdullah Gül University, Kayseri 38080, Turkey; § Department of Physics, Polatlı Faculty of Science and Letters, Ankara Hacı Bayram Veli University, Ankara 06900, Turkey

## Abstract

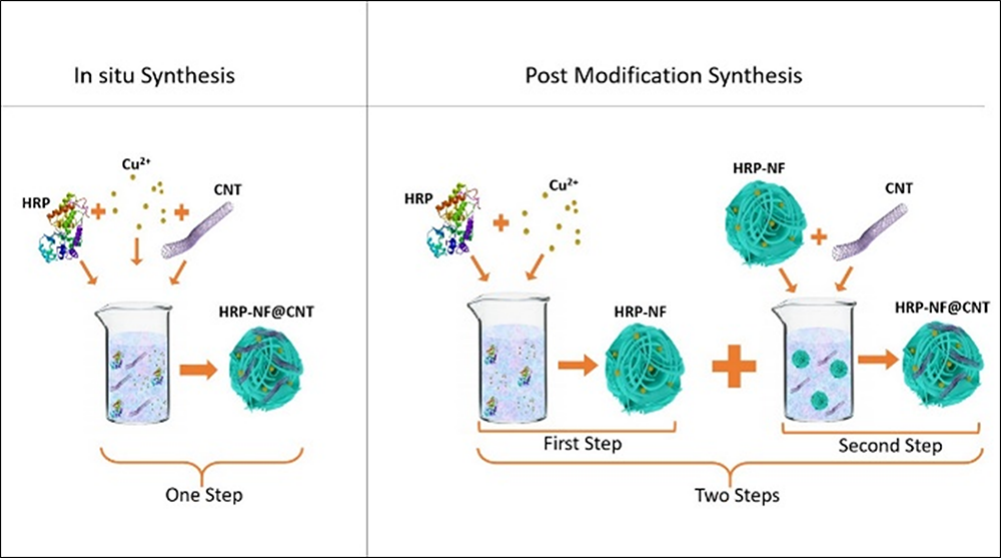

Organic–inorganic hybrid nanoflowers (NFs) consisting of horseradish peroxidase (HRP) and copper II (Cu^2+^) are successfully synthesized with the involvement of carbon nanotubes (CNTs) by in situ and post-modification methods. Catalytic activities of in situ synthesized HRP-NF@CNT (HRP-NF@CNT-Is) and post-modification-synthesized HRP-NF@CNTs (HRP-NF@CNT-Pm) are systematically examined. The 30 mg CNTs incorporated HRP-NF@CNT-Is (HRP-NF@CNT-30Is) exhibits greatly increased catalytic activity and stability toward 3,3′,5,5′-tetramethylbenzidine (TMB), thanks to the synergistic effect between HRP-NF and CNTs and the peroxidase-like activity of CNTs in the presence of hydrogen peroxide (H_2_O_2_). While HRP-NF@CNT-30Is retains almost 85% of its initial activity even after 10 cycles, HRP-NF (without CNTs) loses half of its initial activity at the same experimental conditions. We study how two experimental parameters, the pH values and temperatures, influence the catalytic activity of HRP-NF@CNT-30Is, in addition to the fact that HRP-NF@CNT-30Is is employed to detect the presence of H_2_O_2_ and glutathione (GSH) with colorimetric and spectrophotometric readouts. For instance, HRP-NF@CNT-30Is is used to sensitively detect H_2_O_2_ in the range of 20 to 300 μM with an LOD of 2.26 μM. The catalytic activity of HRP-NF@CNT-30Is is suppressed in the presence of GSH, and then an obvious color change from blue to nearly colorless is observed. Using this strategy, GSH is also sensitively determined in the range of 20–200 μM with an LOD of 11.2 μM. We expect that HRP-NF@CNTs can be used as a promising and novel nanobiocatalyst for various biomedical and industrial applications in the near future.

## Introduction

Enzymes are biomolecules with extraordinary activity, selectivity, and substrate specificity that can catalyze different chemical reactions under normal conditions.^1^ Enzymes are widely used in various fields such as food, pharmaceutical research, textile, and agricultural industries as well as in catalyzing many reactions in in vivo and in vitro systems.^2^ However, the use of free enzymes is troublesome due to the high cost and difficulties in the extraction, separation, and purification steps. They also pose problems in practical applications due to their limited activity and stability under high temperature and extreme pH and in the presence of metal ions and organic solvents.^3^ Therefore, developing efficient immobilization techniques is indispensable for improving enzyme activity and stability.^4^ Recently, flower-shaped nanostructures called nanoflowers (NFs) formed by metal ions and organic molecules have attracted extensive attention owing to their greatly enhanced catalytic activity, higher stability, and reusability compared to free enzymes.^5,6^ The main reasons for the enhanced catalytic activity of enzymes immobilized in NF form can be explained as follows: (i) the high surface area of NFs, which leads to a decrease in mass transfer limitations, (ii) having more active sites in the branch of NFs, and (iii) enhanced local concentration of enzymes in NFs.^6,7^ Given their many advantages, organic–inorganic hybrid NFs offer new opportunities for the growth of the biomedical and biotechnology industries.

Carbon nanotubes (CNTs) are a class of carbon materials that have drawn attention due to their properties such as great chemical stability, high mechanical strength, large surface area, and excellent catalytic activity.^8,9^ The catalytic effect of CNTs originates from their intrinsic properties rather than metal residues. Because of this property, CNTs can be considered as a metal-free catalyst for many biological reactions. For instance, Song et al. reported that CNTs have peroxidase-like activity and can catalyze the oxidation of peroxidase substrate 3,3′,5,5′-tetramethylbenzidine (TMB) in the presence of H_2_O_2_, thereby producing color change.^10^ CNTs have been used as supporting materials for enzyme immobilization due to their large surface area, good adsorption capacity, thermal and chemical features, and good biocompatibility.^11,12^ In particular, enzymes immobilized on CNTs by noncovalent approaches via hydrophobic and π–π interactions have elevated catalytic activity and stability.^13−15^ For instance, Li et al. reported horseradish peroxidase (HRP) immobilization on CNTs via physical adsorption with enhanced catalytic activity, thermal and acid–base stability, and excellent reusability compared to free enzymes.^16^

H_2_O_2_, as a byproduct of oxygen metabolism, has important functions in living organisms.^17^ H_2_O_2_ level is used as the main biomarker of oxidative stress, which is thought to play a role in the development of diseases such as diabetes, cancer, and arteriosclerosis.^18^ Facile and accurate detection methods for colorimetric and quantitative determination of H_2_O_2_ are crucial in medicine, biology, and many other fields.^19,20^ Glutathione (GSH, l-γ-glutamyl-l-cysteinyl-glycine) is a thiol-containing tripeptide molecule that prevents damage to healthy cells caused by reactive oxygen species (ROS), including hydroxyl radicals, superoxide anions, and peroxides.^21^ Glutathione, as an antioxidant, plays an important role in biological systems by regulating the cellular redox balance associated with some serious diseases such as cancer, diabetes, and AIDS.^22^ Abnormal GSH levels are related to decreased immune function and disease progression like heart problems, liver damage, and Alzheimer’s disease.^23,24^ Moreover, GSH as a significant biomarker has a crucial role in biochemical pathways and pathological processes. Detection of the GSH concentration in biological systems is critical in the early diagnosis of diseases.^25−27^ Therefore, development of simple, accurate, and selective analytical procedures is crucial. Recently, the use of colorimetric techniques in GSH detection has attracted attention in terms of being rapid and practical.^26^

In this study, we developed HRP-NF@CNTs with high activity and stability. Briefly, HRP-NF@CNTs were prepared by in situ and post-modification methods. We revealed that the HRP-NF@CNTs prepared by in situ methods showed much enhanced catalytic activity and stability compared to the HRP-NF@CNTs prepared by post-modification methods and only HRP-NF. Additionally, it was observed that the activity of HRP-NF increased with the increase of CNT content because CNTs have peroxidase-like activity and supply more loading sites for HRP. The HRP-NF@CNTs were able to (i) catalyze H_2_O_2_ decomposition to produce hydroxyl radicals (·OH), (ii) oxidize TMB to TMB_ox_, and then (iii) convert the colorless solution to blue through peroxidase reaction. The existence of glutathione, which has an inhibition effect on HRP, suppresses the catalytic activity of HRP-NF@CNTs leading to conversion of TMB_ox_ to TMB.^28^ After optimizing parameters of reaction such as pH, incubation time, and temperature, the change of absorbance was linearly correlated with the GSH concentration.

## Experimental Methods

### Synthesis of CNTs

CNTs were synthesized with the chemical vapor deposition (CVD) method in a 1″-diameter catalytic CVD furnace. Details of the synthesis are described in elsewhere.^29−31^ Briefly, CNT synthesis was performed on a SiO_2_ substrate coated with 1/10 nm Fe/Al_2_O_3_, as a catalyst layer. Fe catalysts were reduced in 100/25 sccm H_2_/Ar at 500 °C for 5 min before the synthesis. Subsequently, CNT synthesis was started by adding 25 sccm C_2_H_4_ to the gas mixture at 800 °C. A 30 min synthesis resulted in a 1.5 mm-long vertically aligned CNT forest. After the synthesis, the CNT forest was removed from the substrate with a razor. The CNT forest was then sonicated with a probe sonicator in phosphate-buffered saline (PBS, pH 7.4) solution in order to obtain homogenously dispersed CNTs.

### Synthesis of HRP-NF

The synthesis of HRP-NF was completed by following reported studies.^7,32^ Briefly, a certain amount of HRP was mixed into 10 mM PBS buffer (pH 7.4), and then freshly prepared 0.8 mM CuSO_4_ was added into that mixture. The final mixture was vigorously shaken with a vortex for homogeneity, and then it was incubated for 72 h at 25 °C. HRP reacted with the Cu_3_(PO_4_)_2_ crystals formed as primary crystals in the nucleation step to produce HRP-Cu_3_(PO_4_)_2_ nanopetals in the growth step. The sticking of these nanopetals induces formation of whole-flower-shaped hybrid nanostructures called “nanoflower” (NF).^7,32^

### Synthesis of HRP-NF@CNTs

#### In Situ Synthesis of HRP-NF@CNTs

10 and 30 mg of CNTs were added into 1 L of PBS (10 mM, pH 7.4) and dispersed by ultrasonication for 30 min. 20 mg of HRP was added to these solutions and stirred for 2 h. Subsequently, an aqueous solution of CuSO_4_·5H_2_O was added to the resulting mixtures and incubated for 12 h at room temperature (25 °C) without disturbing. After the incubation, the mixture was centrifuged and the obtained precipitates were dried at 40 °C. The samples containing the 10 and 30 mg CNTs were referred to as HRP-NF@CNT-10Is and HRP-NF@CNT-30Is, respectively.

#### Post-Modification Synthesis of HRP-NF@CNTs

For synthesis of HRP-NF@CNTs with post modification, 10 and 30 mg of CNTs were added into 1 L of deionized water and dispersed by ultrasonication for 30 min. The previously synthesized HRP-NF was added to these mixtures and vigorously stirred for 3 h followed by centrifugation for 10 min at 5000 rpm. The precipitates were dried at 40 °C. The samples containing the 10 and 30 mg of CNTs were referred to as HRP-NF@CNT-10Pm and HRP-NF@CNT-30Pm, respectively.

#### Catalytic Activity of HRP-NF@CNTs

The peroxidase activities of hybrid structures were determined by monitoring the oxidation of TMB. TMB (100 μL, 15 mM) was added to the mixture, containing a catalyst (375 μL, 4 mg/mL) and H_2_O_2_ (15 μL, 10 mM) in sodium acetate (NaAc) buffer solution (pH 3.5, 10 mM). The catalytic oxidation of TMB was monitored by recording absorbance values at 652 nm. The effects of pH and temperature on the peroxidase activity of the hybrid structures were also investigated by changing the pH (3.5–9.5) and temperature (20–60 °C).

#### Steady-State Kinetic Analysis of HRP-NF and HRP-NF@CNT-30Is

The steady kinetic analysis of HRP-NF@CNT-30Is and HRP NF was carried out in a 1.5 mL NaAc buffer (10 mM, pH 3.5) containing HRP-NF@CNT-30Is or HRP NF (1 mg mL^–1^), TMB (1 mM), and H_2_O_2_ with various concentrations (0.02 mM–0.9 mM). The absorbance values were recorded at 652 nm for 20 min. The steady-state reaction rates were calculated by the changing of the slope of absorbance with time. The kinetic parameters were derived from using the Michaelis–Menten equation:
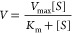
1

*V* is the initial reaction velocity, *V*max is the maximum reaction velocity, [*S*] is the substrate concentration, and *K*_m_ is the Michaelis–Menten constant.

#### Reusability of HRP-NF@CNTs

The reusability of HRP-NF and HRP-NF@CNT-30Is was investigated for 10 cycles by catalyzing TMB in the presence of H_2_O_2_ under optimum reaction conditions. Each time after the reaction, the NFs were centrifuged and washed with water for removing residual substrates or products and then exposed to air to dry. This procedure was repeated after each reaction.

### Detection of H_2_O_2_ and GSH

Different amounts of H_2_O_2_ were added to NaAc buffer solution containing 1 mg/mL of catalyst and 1 mM TMB. The mixture was incubated at 40 °C for 30 min. After removing the catalyst by centrifugation, the absorbance intensity of the supernatant at 652 nm was recorded for quantitative detection of H_2_O_2_.

For GSH detection, different concentrations of GSH were mixed with 375 μL of catalyst (4 mg/mL) and 150 μL of H_2_O_2_ (1 mM) in pH 3.5 NaAc buffer and incubated for 30 min to ensure inhibition and reduction of HRP-NF@CNT-30Is by GSH. Then, 100 μL of TMB was added to the solution and incubated for 15 min to ensure completion of the reaction. HRP-NF@CNT-30Is was removed from the reaction solution by centrifugation. The absorbance values of the supernatant at 652 nm were used as a function of GSH concentration.

To investigate the selectivity of the current method toward H_2_O_2_ and GSH detection, determined volumes of stock solutions of different types of interferences were added to HRP-NF@CNT-30Is + TMB + H_2_O_2_ solution to reach the final concentration of 0.5 mM and then the peak intensity at 652 nm was measured after incubation for 30 min.

## Results and Discussion

### Characterization of HRP-NF@CNTs

Organic–inorganic hybrid NFs were generated by coordination reaction between functional groups (especially amine groups) of organic molecules and metal ions (especially Cu^2+^ ions) in PBS buffer. Herein, we employed two synthesis methods, in situ and post-modification, to fabricate HRP-NF@CNTs. CNTs acted as an external support for both HRP and HRP-NF immobilizations in in situ synthesis, but they supported only HRP-NF in post-modification synthesis. Additionally, CNTs contribute to enhanced catalytic activity and increase stability due to their intrinsic peroxidase-like activity and immobilization of HRP. In the preparation of HRP-NF@CNTs by in situ synthesis and post-modification, we hypothesize that while some portion of HRP binds to CNTs, accessible amide groups (−NH_2_) on the backbone of the HRP react with Cu^2+^ in PBS to form complexes. Then, we successfully synthesized HRP-NF@CNTs by in situ synthesis at 12 h. However, for the synthesis of HRP-NF@CNTs with post-modification, the HRP-NF formed at 72 h and then combined with CNTs.

**Figure 1:**
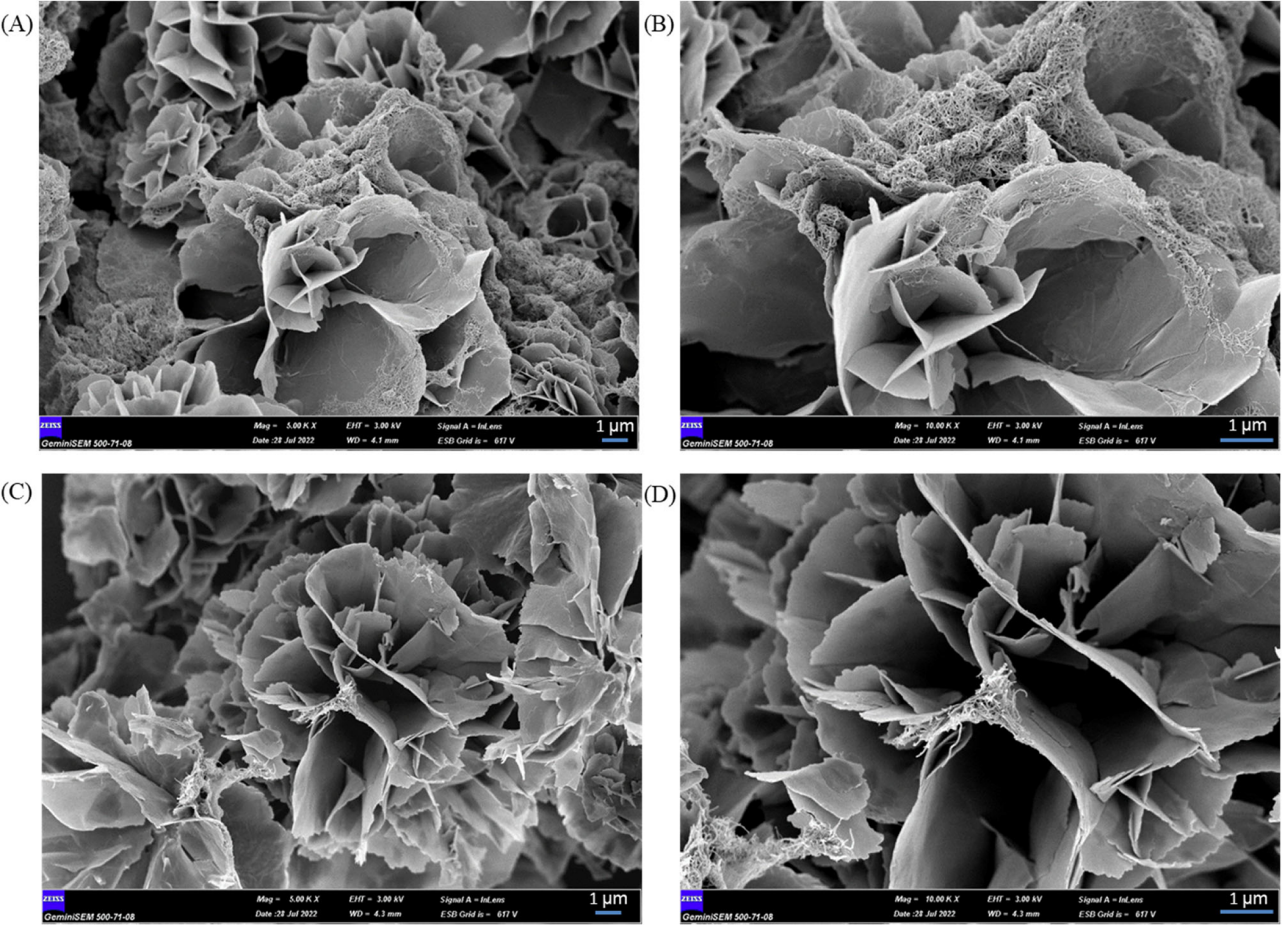
SEM images of HRP-NF@CNTs synthesized by the in situ method as a function of incubation times of (A, B) 12 h and (C, D) 24 h. SEM images in (B) and (D) are the magnified views of (A) and (C), respectively (A and C: magnification at 5,000×, B and D: magnification at 10,000×).

SEM images in Figure 1 show the morphology of HRP-NF@CNTs synthesized by the in situ method at 12 and 24 h of incubation. The morphology of HRP-NF@CNTs was similar to that of previously reported HRP-NF.^7,33^ The addition of CNTs did not change the morphology of HRP-NF, but CNTs allowed the NFs to hold together, making them more compact structures (Figure 1). According to Figure 1, CNTs were located between NFs and on petals. Furthermore, CNTs enabled the formation of HRP-NF in a short time (12 h) compared to literature.^32−34^ It was observed that NF structures were formed at the incubation times of 12 and 24 h. The synthesis of HRP-NFs in one-step with rapid formation was attributed to the use of CNTs as a support.

**Figure 2:**
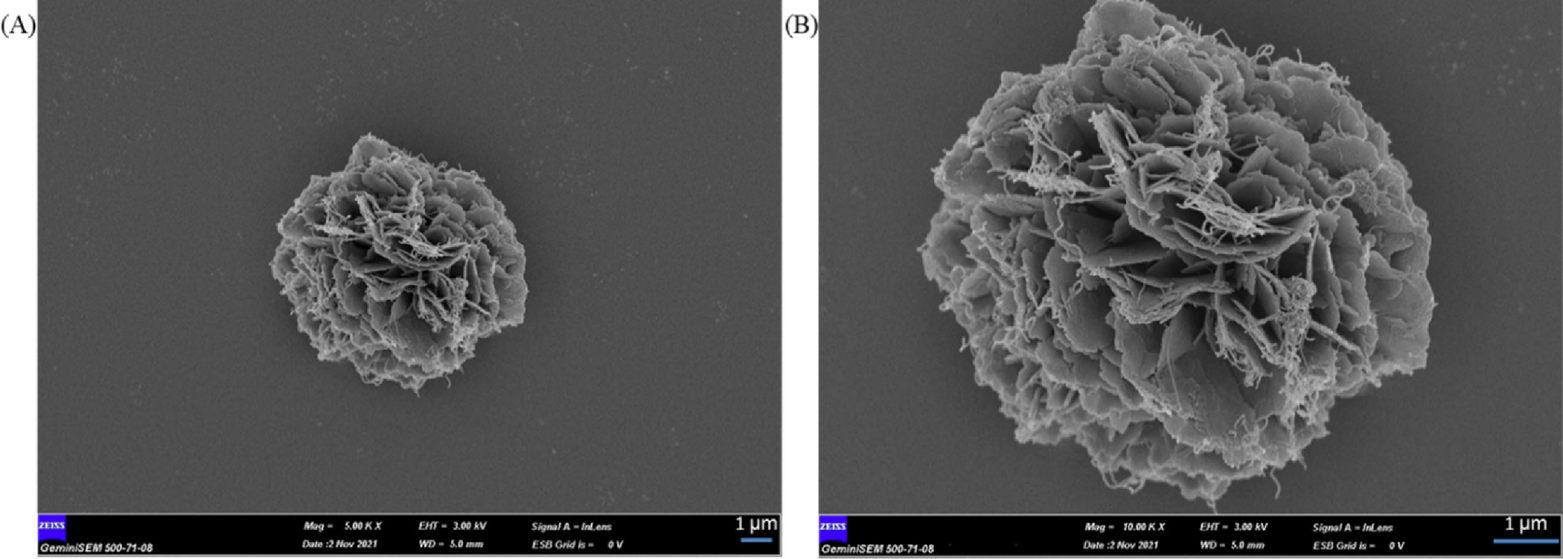
(A, B) SEM images of HRP-NF@CNTs synthesized by the post-modification method at different magnifications (A: magnification at 5,000×, B: magnification at 10,000×).

SEM images of HRP-NF@CNTs synthesized using the post-modification method display their morphologies with different magnifications shown in Figure 2A,B. As seen in Figure 2, flower petals of the HRP-NF were covered by the CNTs and there is no change in the morphology of the HRP-NF.

The HRP-NF@CNTs were elementally analyzed using energy-dispersive X-ray (EDX) spectroscopy. As shown in Figure S1, carbon (C) was attributed to HRP and CNTs. The existence of nitrogen (N), oxygen (O), phosphorous (P), and copper (Cu) confirmed the successful synthesis of HRP-NF@CNTs.

**Figure 3:**
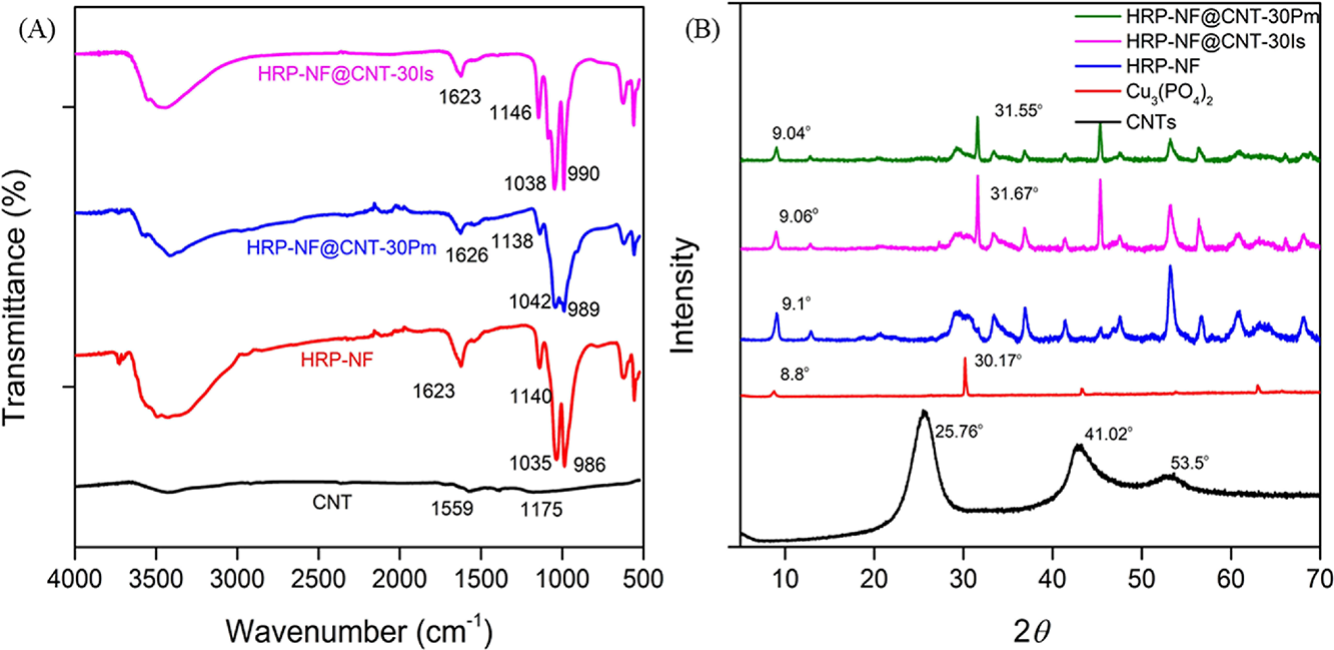
(A) FTIR spectra of HRP-NF@CNT-30Is, HRP-NF@CNT-30Pm, HRP-NF, and CNTs. (B) XRD patterns of HRP-NF@CNT-30Pm, HRP-NF@CNT-30Is, HRP-NF, Cu_3_(PO_4_)_2_, and CNTs.

FTIR analysis was performed to verify the molecular structure of HRP-NF@CNTs. Hybrid structures gave peaks at almost the same wavenumbers. As shown in Figure 3A, IR bands at 1035 and 986 cm^–1^ are attributed to PO_4_^3–^ vibrations of Cu_3_(PO_4_)_2_ and the peaks at 1623 cm^–1^ represent the amide carbonyl group in HRP.^38,39^ Based on these results, HRP was successfully immobilized to Cu_3_(PO_4_)_2_. Moreover, the chemical structures of the HRP-NF@CNTs synthesized by the in situ and post-modification methods have not changed, but since the FTIR peaks of the HRP-NF are dominant, the specific peaks of the CNTs cannot be seen in HRP-NF@CNTs.

XRD patterns in Figure 3B demonstrate the crystallographic structures of CNTs, HRP-NF, HRP-NF@CNT-30Is, and HRP-NF@CNT-30Pm. The positions of all diffraction peaks of HRP-NF, HRP-NF@CNT-30Is, and HRP-NF@CNT-30Pm matched well with those of Cu_3_(PO_4_)_2_·3H_2_O crystals (JCPDS card 22-0548), indicating that the main constituent of all samples was Cu_3_(PO_4_)_2_.^5,7^ Additionally, diffraction peaks of CNTs at 25.76, 41.02, and 53.50° correspond to (002), (100), and (004) of CNTs that are consistent with the JCPDS card (75-1621) of graphite.^35^

### Peroxidase Activity of HRP-NF@CNTs

**Figure 4:**
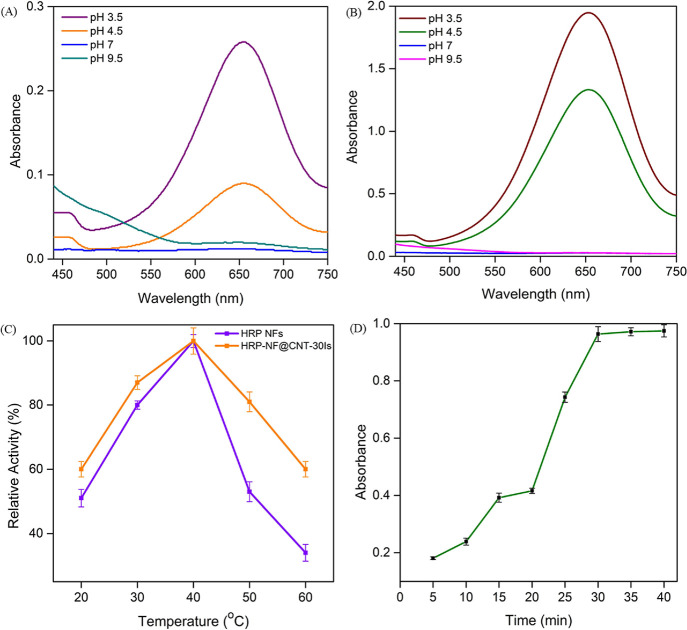
Optimum reaction conditions: pH varying activities of (A) HRP-NF and (B) HRP-NF@CNT-30Is. (C) Temperature varying activities of HRP-NF and HRP-NF@CNT-30Is. (D) Reaction time effect on the catalytic activity of HRP-NF@CNT-30Is.

To investigate the peroxidase activities of the catalyst materials, peroxidase substrates were oxidized in the presence of H_2_O_2_ and the responses were monitored using a UV–vis spectrometer. The catalytic activities of the catalyst materials are highly dependent on reaction parameters including concentration of the catalyst, pH, temperature, and incubation time. We investigated the effect of pH values (pH: 3.5, 4.5, 7, and 9.5) on the catalytic activities of HRP-NF and HRP-NF@CNT-30Is toward TMB used as a model substrate. As displayed in Figure 4A,B, HRP-NF@CNT-30Is and HRP-NF showed maximum catalytic activity by oxidizing TMB at pH 3.5. When the reaction pH increased especially reaching 7 and 9.5, the activities of HRP-NF and HRP-NF@CNT-30Is were almost completely lost due to much higher negative charges at the surface of the HRP (its isoelectric point: 5.5). According to Figure 4C, the optimum reaction temperature was determined at 40 °C for HRP-NF and HRP-NF@CNT-30Is. It is worth mentioning that HRP-NF@CNT-30Is exhibited much high tolerance to high temperatures compared to HRP-NF. We think that the presence of CNTs increases the stability of HRP-NF@CNT-30Is. Moreover, we investigated the effect of reaction time on the catalytic activity of HRP-NF@CNT-30Is (Figure 4D). In the presence of 200 μM H_2_O_2_, the intensity of the blue color by the oxidation of TMB displayed negligible change after 30 min. Therefore, the optimum reaction time was determined to be 30 min.

In order to understand the reaction catalysis mechanism of CNTs, radical capturing experiments were performed. Here, tryptophan as the quencher of singlet oxygen molecules (^1^O_2_) and isopropanol as that of ·OH were added into the system of CNTs+H_2_O_2_ + TMB.^40,41^ As shown in Figure S2, in the presence of tryptophan, the absorbance intensity of the solution does not decrease significantly, whereas decreased absorbance value with the addition of isopropanol can be observed, which indicates that ·OH radicals are generated from H_2_O_2_ in the reaction process (eq 2).
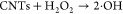
2

**Figure 5:**
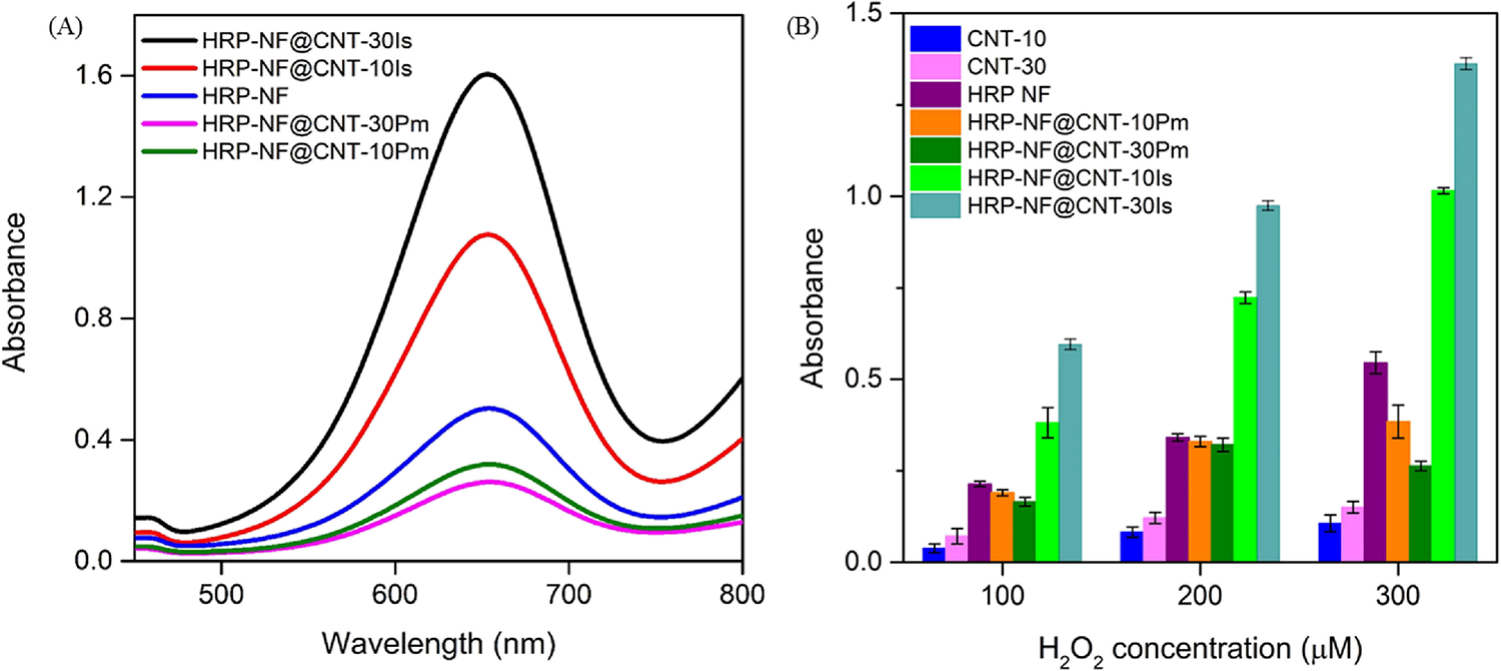
(A) Activities of HRP-NF and HRP-NF@CNTs. (B) Activity comparison of HRP-NF@CNTs, HRP-NF, and CNTs in different H_2_O_2_ concentrations.

After determining the optimum reaction conditions, the catalytic activities of the HRP-NF@CNTs synthesized by the in situ and post-modification methods were compared (Figure 5A). The order of peroxidase activities of the nanocatalysts was found as follows: HRP-NF@CNT-30Is > HRP-NF@CNT-10Is > HRP-NF > HRP-NF@CNT-10Pm > HRP-NF@CNT-30Pm.

It was concluded that the activity of HRP-NF@CNTs synthesized by the in situ method is higher than that synthesized by post-modification. Herein, we hypothesized possible factors for the higher activity of HRP-NF@CNT-Is: (i) HRP-NF was synthesized between CNT molecules without blocking the active side of HRP. (ii) Because the HRP enzyme was immobilized on CNTs during the initial stages of NF synthesis, its catalytic activity may have increased. (iii) CNTs may induce favorable conformational changes of HRP. Additional factors were (iv) the high surface area of HRP-NF@CNTs and (v) the cooperative effect between HRP-NF and CNT. As shown in Figure 5B, only CNTs exhibited intrinsic peroxidase-like activities. The activity of HRP-NF@CNT-30Is is higher than that of HRP-NF@CNT-10Is. It states that CNTs with peroxidase-like activity cause more oxidation of TMB, which is an indicator of increased activity. Conversely, activities of HRP-NF@CNT-30Pm and HRP-NF@CNT-10Pm are lower than that of HRP-NF. We claim that (i) because HRP was surrounded by CNTs as shown in Figure 2B, CNTs may block the active side of HRP and the substrate cannot diffuse the active center of HRP, and (ii) unfavorable conformation may occur between HRP-NF and CNTs, both of which may hinder access of the substrates to the active center.^7,32^

### Steady-State Kinetic Analysis of HRP-NF and HRP-NF@CNT-30Is

**Figure 6:**
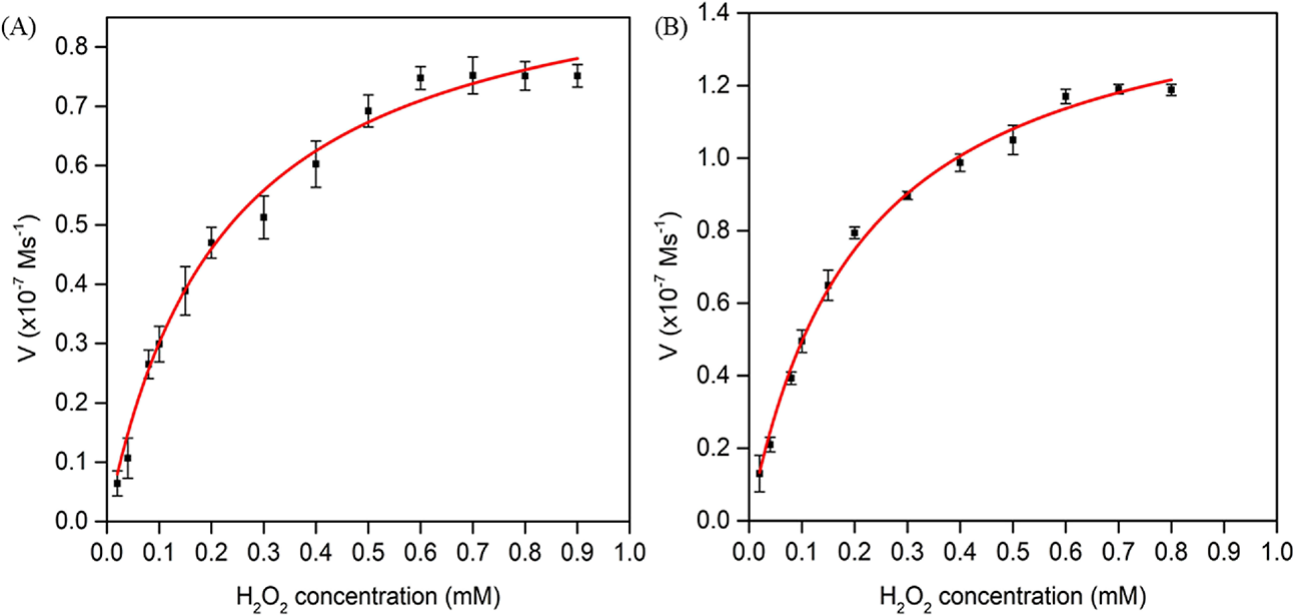
Kinetic analysis according to the Michaelis–Menten model. (A) HRP-NF and (B) HRP-NF@CNT-30Is.

To investigate the catalytic behavior of HRP-NF@CNT-30Is and HRP-NF, steady-state kinetic parameters were determined by varying the concentrations of H_2_O_2_. Typical Michaelis–Menten curves showing the relationship between substrate concentration and initial reaction rate were obtained (Figure 6A,B). *K*_m_ and *V*_max_ values of the HRP-NF and HRP-NF@CNT-30Is displayed in Table 1 were calculated by using the Lineweaver Burk plot (Figure S3). A low *K*_m_ value demonstrates a higher affinity of HRP against H_2_O_2_ and vice versa.^42^ Compared to free HRP, HRP NF and HRP-NF@CNT-30Is had a stronger affinity for H_2_O_2_. It can be confirmed that the crystal structure of NF did not hinder the free diffusion of substrate molecules to the active site of enzymes. Moreover, especially the *V*_max_ value of HRP-NF@CNT-30Is was higher than those of free HRP and HRP-NF, which indicates superior TMB oxidation and enzymatic activity (Figure 6A,B). The *K*_m_ and *V*_max_ values supported the strategy to improve the catalytic performance of HRP-NF by combining with CNTs. This might be due to the synergistic effect of entrapped HRP molecules and CNTs within the hierarchical structure of HRP-NF.^43^

**Table 1:** Michaelis–Menten Constants of HRP-NF, HRP-NF@CNT-30Is, and HRP

catalyst	*K*_m_ (mM)	*V*_max_ (10^–7^ M s^–1^)	ref
HRP-NF	0.220	0.975	this work
HRP-NF@CNT-30Is	0.205	1.527	this work
HRP	3.70	0.871	(42)
			

**Figure 7:**
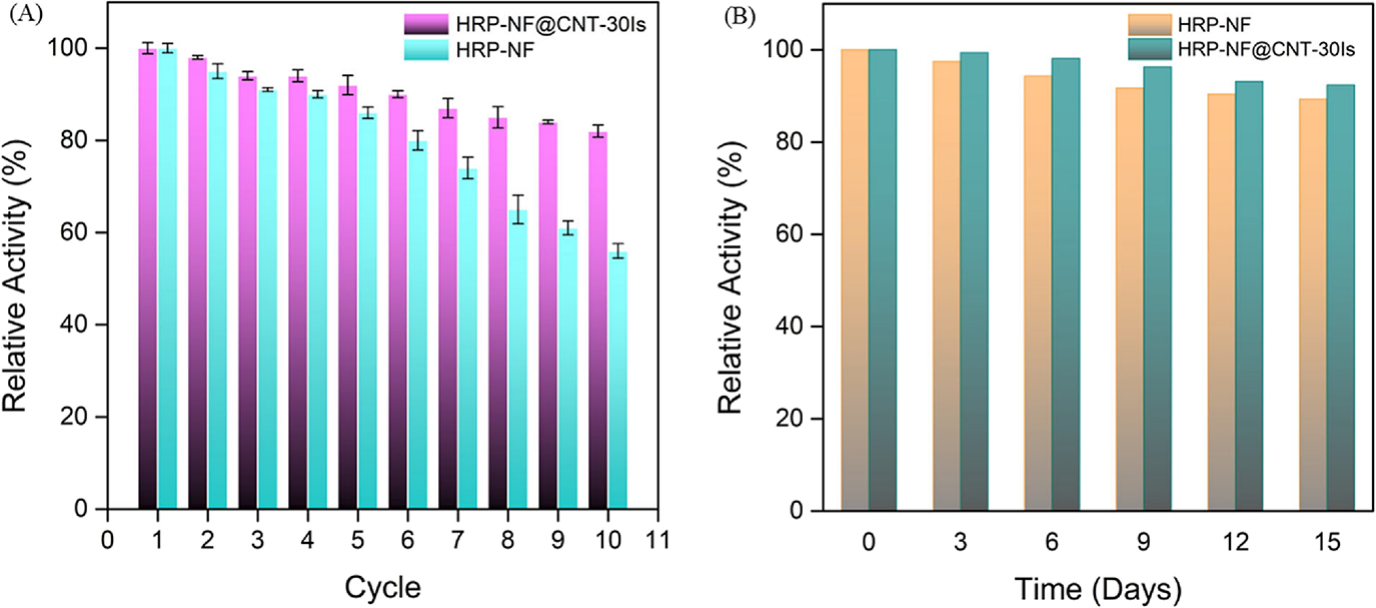
(A) Reusability and (B) storage stability of HRP-NF and HRP-NF@CNT-30Is.

The reusability of HRP-NF and HRP-NF@CNT-30Is was also investigated to evaluate their stability (Figure 7A). After 10 cycles, while HRP-NF lost almost 50% of its initial activity, HRP-NF@CNT-30Is retained almost 85% of its initial activity. These results demonstrated that incorporation of CNTs into NFs enables higher operational stability.

In order to investigate the long-term enzyme storage stability of HRP-NF and HRP-NF@CNT-30Is in the solid state, enzyme activities were measured for 15 days with an interval of 3 days. The activity value obtained on the first day was accepted as 100%. As displayed in Figure 7B, HRP-NF and HRP-NF@CNT-30Is retained about 89.3 and 92.4% of their initial activity at room temperature for 15 days, respectively. These showed that superior reusability and long storage stability of HRP-NF@CNT-30Is can be advantageous for various applications.

### H_2_O_2_ Detection

**Figure 8:**
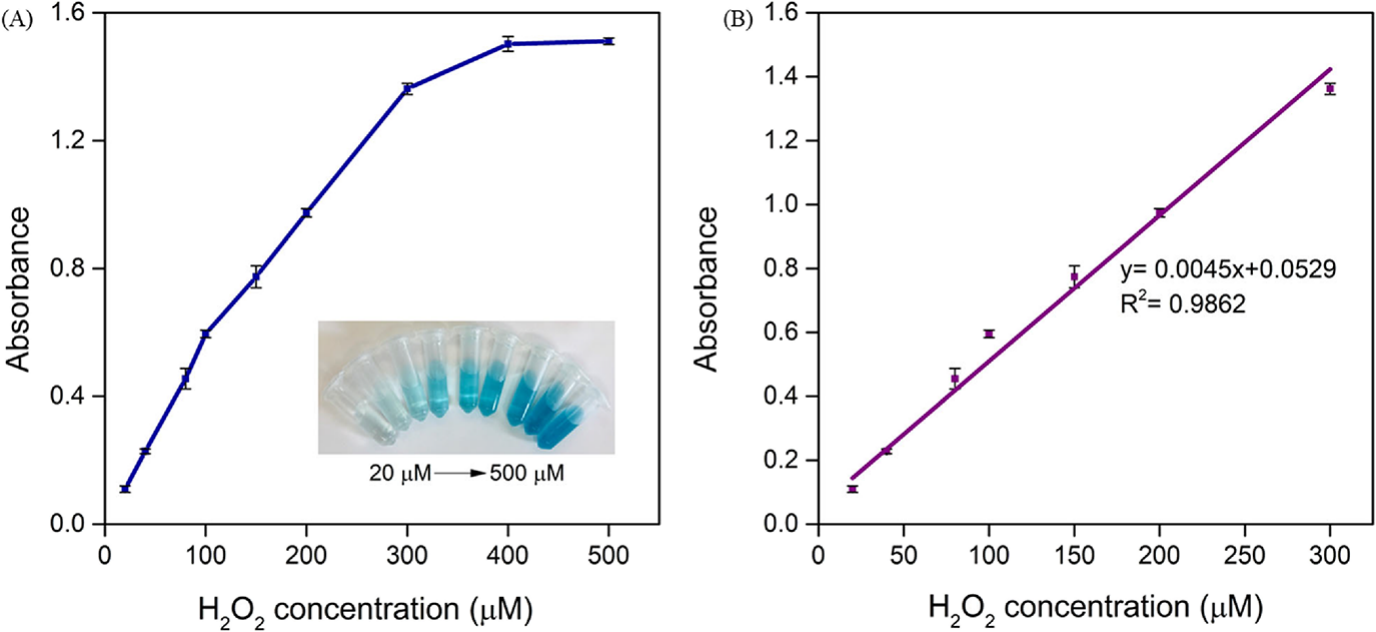
(A) Change in absorbance of HRP-NF@CNT-30Is based on the TMB-H_2_O_2_ assay at 652 nm. Inset: corresponding photograph of the samples. (B) Linear calibration plot of the H_2_O_2_ detection between 20 and 300 μM.

Based on the superior peroxidase activity of HRP-NF@CNT-30Is, a sensitive, simple, and convenient colorimetric method for H_2_O_2_ detection was constructed. It was noted that the absorbance at 652 nm was gradually enhanced as the H_2_O_2_ concentration increased from 20 to 500 μM with a linear response obtained between 20 and 300 μM (*y* = 0.0045*x* + 0.0529, *R*^2^ = 0.9862) (Figure 8A,B). Furthermore, the color change from light to deep blue of the mixed solutions can be discerned with the naked eye (inset in Figure 8A). The limit of detection was calculated to be 2.26 μM based on the equation of LOD = 3σ/*s*, where σ is the standard deviation of blank samples and *s* is the slope of the calibration curve. As compared to previously developed methods (Table S1), the detection limit value of HRP-NF@CNT-30Is is smaller, indicating greater sensitivity to H_2_O_2_ and providing an alternative way for detection of H_2_O_2_.

Selectivity analysis is important for the newly developed sensor systems. The selectivity of HRP-NF@CNT-30Is toward the detection of H_2_O_2_ was evaluated after adding 150 μL (5 mM) of glycine (Gly), cysteine (Cys), valine (Val), glutamic acid (Glu), urea, dopamine, epinephrine, ascorbic acid, glucose, Fe^2+^, Zn^2+^, and Cu^2+^ as interferences into the TMB + H_2_O_2_+ HRP-NF@CNT-30Is reaction system, and the absorbance values were recorded at 652 nm. As shown in Figure 10A, the absorbance of H_2_O_2_ was higher than that of other interferences and the color can be distinguished by the naked eye, although their concentration is threefold higher than that of H_2_O_2_, suggesting that the studied molecules and ions had no interfering effect.

### GSH Detection

Herein, according to decolorization reaction of oxidation products of TMB (TMB_ox_), GSH was quantitatively analyzed using a colorimetric analytical method through peroxidase active HRP-NF@CNT-30Is. The inhibitory mechanism of GSH can be explained by two possible factors: (i) GSH has been reported to inhibit the action of HRP.^28^ (ii) As an antioxidant, GSH can quench the radical cation (·OH), leading to prevention of TMB oxidation.^36,37^ At first, we optimized the detection conditions. Since the maximum activity of HRP-NF@CNT-30Is occurred in NaAc buffer of pH 3.5 at 40 °C, the pH and temperature of the reaction solution were chosen accordingly. When the concentration of H_2_O_2_ is too high, there can be an insignificant difference in color change as the consumption of GSH is too small, affecting the accuracy of detection. When the concentration of H_2_O_2_ is too low, a small amount of GSH can be detected due to the low production of ·OH. Thus, the concentration of H_2_O_2_ was selected as 100 μM.

**Figure 9:**
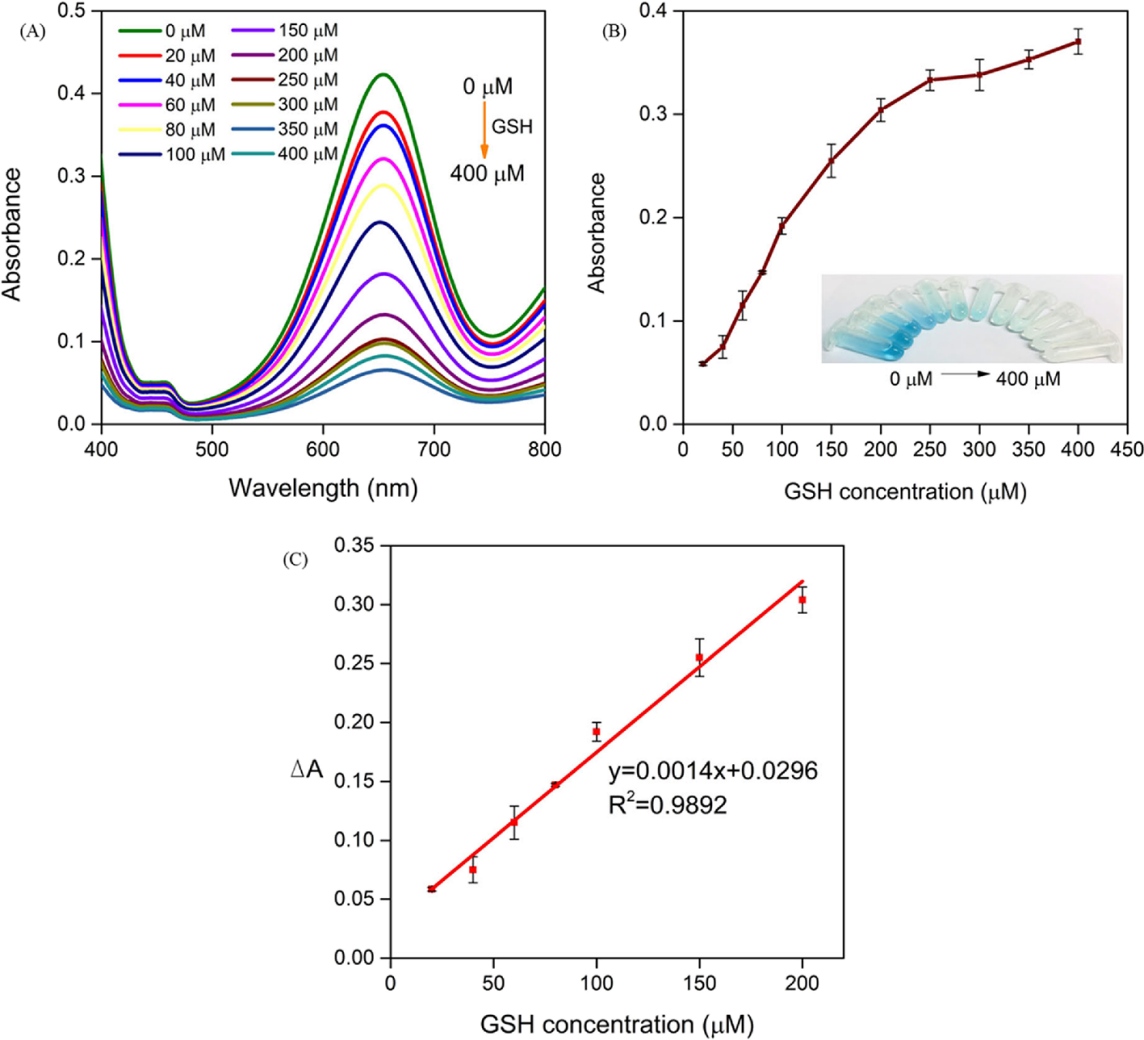
(A, B) Change in absorbance of HRP-NF@CNT-30Is based on the TMB-H_2_O_2_ assay with increasing GSH concentration at 652 nm. Inset: corresponding photograph of the samples. (C) Linear calibration plot for the determination of GSH using the HRP-NF@CNT-30Is-based assay.

For the colorimetric detection of GSH, a mixture containing H_2_O_2_, GSH, and HRP-NF@CNT-30Is was incubated for 30 min to complete the reduction of ·OH and inhibited the action of HRP by GSH. Then, TMB was added to the mixture and incubated for another 15 min to complete the reaction. At the end of the reaction, GSH reduced TMB_ox_ to TMB and simultaneously oxidized itself into glutathione disulfide (GSSG). As shown in Figure 9A,B, the absorbance intensity of TMB_ox_ at 652 nm decreased and the blue color faded gradually with the elevated dosage of GSH (Figure 9B). The difference in the absorbance value, Δ*A* (Δ*A* = *A*_blank_ – *A*_GSH_, where *A*_blank_ and *A*_GSH_ are the absorbance at 652 nm in the absence and presence of GSH, respectively), reached a plateau when the concentration of GSH was up to 400 μM, and a linear relationship is seen between 20 and 200 μM (Δ*A* = 0.0014*x* + 0.0296, *R*^2^ = 0.9892). LOD was calculated as 11.2 μM (Figure 9C). Compared with other methods for GSH detection (Table S2), the developed system can show a lower LOD. Therefore, the proposed method can be a candidate for the practical colorimetric sensing of GSH in various samples.

**Figure 10:**
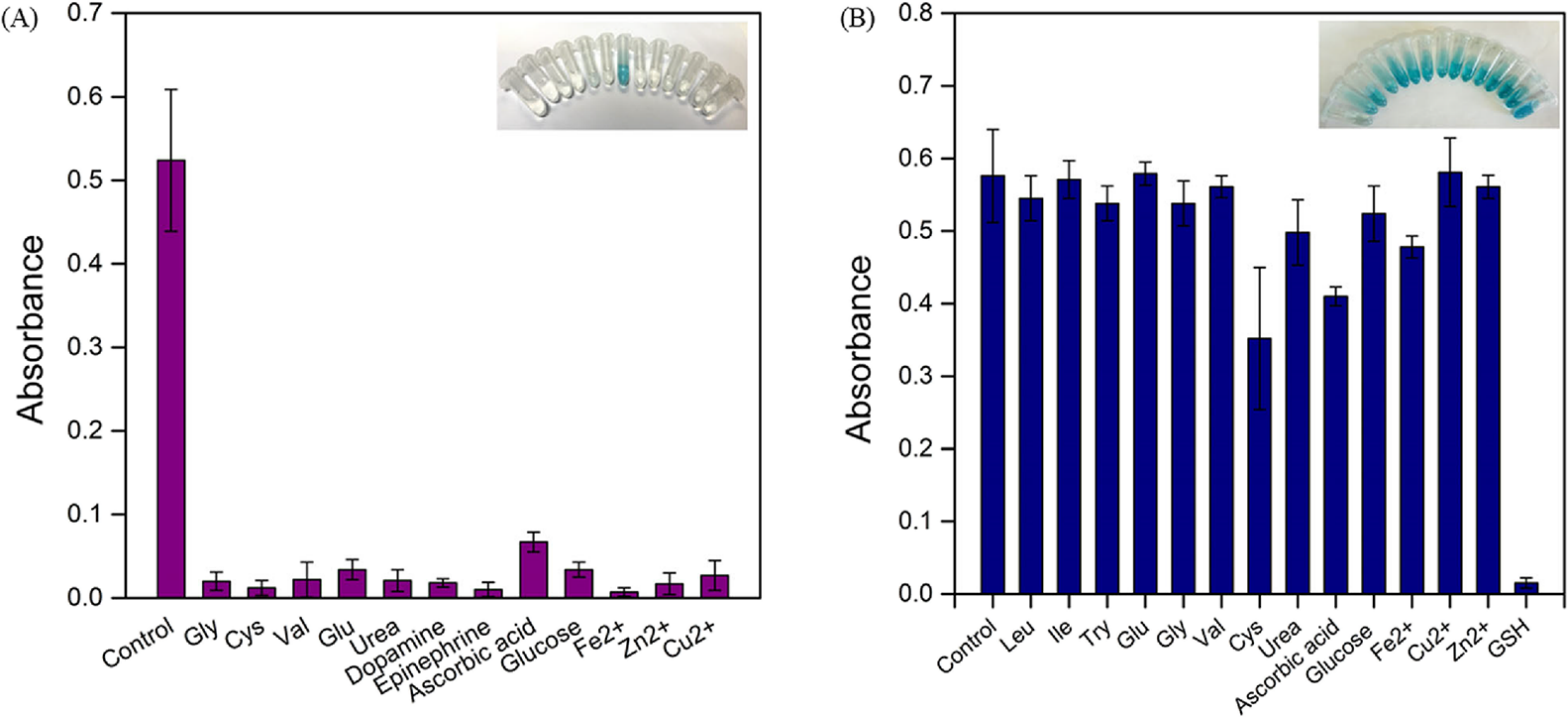
Selectivity of (A) H_2_O_2_ and (B) GSH with the various interferences using HRP-NF@CNT-30Is.

The selectivity study of HRP-NF@CNT-30Is toward GSH detection was carried out to investigate potential interferences of ions (Fe^2+^, Zn^2+^, and Cu^2+^); amino acids including leucine (Leu), isoleucine (Ile), arginine (Arg), tryptophan (Try), Val, Glu, Gly, and Cys; and biomolecules (urea, ascorbic acid, glucose). Figure 10B shows the absorbance values at 652 nm after adding interfering agents, and it was found that there was an inconsiderable change in absorbance at 652 nm in the presence of interferences except cys, ascorbic acid, Fe^2+^, and GSH. The addition of cys, ascorbic acid, and Fe^2+^ led to an approximately 30–40% decrease in absorbance values, but GSH induced a 97% decrease in absorbance signal. The reason may be its high reducing ability to TMBox and that it oxidizes itself into GSSG.^44^ These results clearly showed that HRP-NF@CNT-30Is offers superior selectivity toward GSH.

### H_2_O_2_ and GSH Detection in the Spiked Serum Sample

To investigate the feasibility of the proposed sensor system, simulated human serum was used for the detection of H_2_O_2_ and GSH. The HRP-NF@CNT-30Is + TMB-based sensing system was inspected in the simulated human serum, which was prepared according to literature.^45^ The serum was spiked with additional known concentrations of H_2_O_2_ (40, 80, and 120 μM) and GSH (50, 100, 150), and the analyses were performed as described above. Table 2 demonstrates the analytical performance of HRP-NF@CNT-30Is on the determination of H_2_O_2_ and GSH in simulated serum samples. The recoveries of H_2_O_2_ and GSH were between 97.1 and 102.15%, and the relative standard deviation values are less than 5%, suggesting that HRP-NF@CNT-30Is can be used as a promising nanobiocatalyst for bioanalytical applications.

**Table 2:** Analytical Performance of the Determination of H_2_O_2_ and GSH in Spiked Human Serum Samples

materials	spiked amount (μM)	found amount (μM)	recovery (%)	RSD (%)
H_2_O_2_	40	39.32	98.3	4.83
	80	81.72	102.15	3.64
	120	117.54	97.95	4.11
GSH	50	48.82	97.64	2.78
	100	97.1	97.1	4.32
	150	152.4	101.6	4.44

## Conclusions

In summary, HRP-NF@CNTs were successfully fabricated through the biomineralization process with involvement of CNTs. The resulting HRP-NF@CNTs synthesized by the in situ method exhibited outstanding peroxidase activity and stability due to synergistic integration of both CNTs and Cu_3_(PO_4_)_2_ crystals. HRP-NF@CNT-30Is displayed excellent stability even after 10 cycles compared to HRP-NF. The kinetic parameters of HRP-NF@CNT-30Is were improved compared to free HRP. Outstandingly, the *K*_m_ value of free HRP was reduced 18.05-fold for HRP-NF@CNT-30Is. Furthermore, HRP-NF@CNT-30Is was used for colorimetric and spectrophotometric detection of H_2_O_2_ and GSH with a low LOD and a wide linear range. The proposed colorimetric and spectrophotometric detection system shows an excellent selectivity over different interferences such as biomolecules, amino acids, and ions. These results demonstrate that HRP-NF@CNTs having high activity and stability can be used in clinical and biomedical applications in the near future. This study will provide new opportunities for the better design of other materials with improved performance.

## Supporting Information Available

EDX analysis of HRP-NF@CNT-30Is, absorbance spectrum of the HRP-NF@CNT-30Is-TMB-H_2_O_2_ system in the presence of isopropanol and tryptophan, Lineweaver Burk plot for HRP-NF and HRP-NF@CNT-30Is, list of linear ranges and detection limits for H_2_O_2_ and GSH of various materials (PDF)
