# Supplementary material for: In Situ Synthesis of Horseradish Peroxidase Nanoflower@Carbon Nanotube Hybrid Nanobiocatalysts with Greatly Enhanced Catalytic Activity
Source: Langmuir. 2023 Mar 21;39(13):4819–28. doi: 10.1021/acs.langmuir.3c00260 (PMC10077815; doi:10.1021/acs.langmuir.3c00260)
Supplement: Supplementary file 1 — la3c00260_si_001.pdf [file la3c00260_si_001.pdf]

Supplemental Files

In situ Synthesis of Horseradish Peroxidase-Nanoflower@Carbon Nanotube Hybrid Nanobiocatalysts with Greatly Enhanced Catalytic Activity

Seyma Dadi[1,2], Nimet Temur[1], O. Tolga Gul[3], Vedat Yilmaz[1] and Ismail Ocsoy[1,]*

[1]Department of Analytical Chemistry, Faculty of Pharmacy, Erciyes University, 38039 Kayseri, Turkey

[2]Department of Nanotechnology Engineering, Abdullah Gül University, Kayseri 38080, Turkey

[3]Department of Physics, Polatlı Faculty of Science and Letters, Ankara Hacı Bayram Veli University, Ankara 06900, Turkey

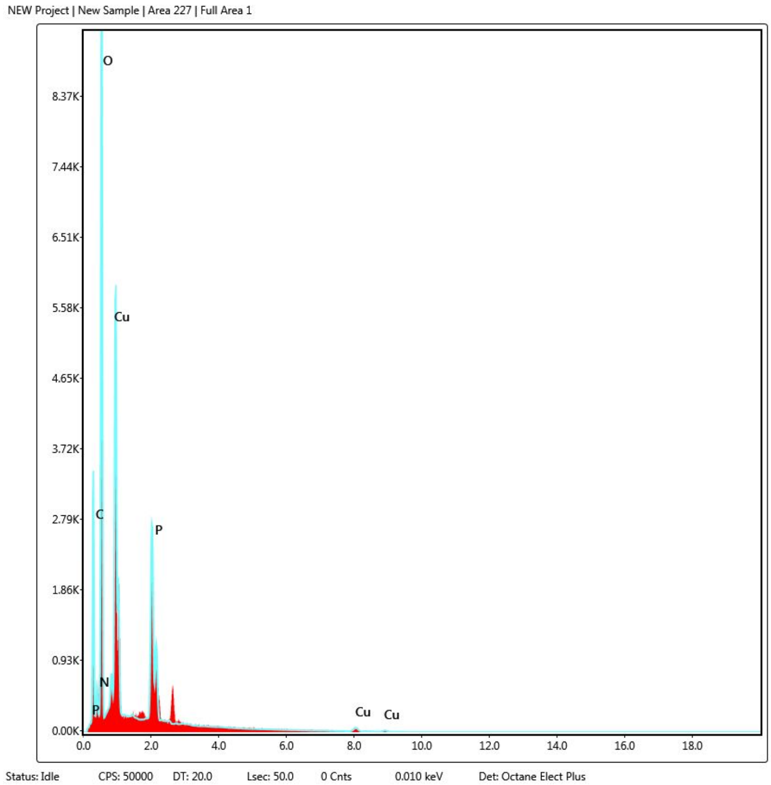

Figure S1. EDX analysis of HRP-NF@CNT-30Is.

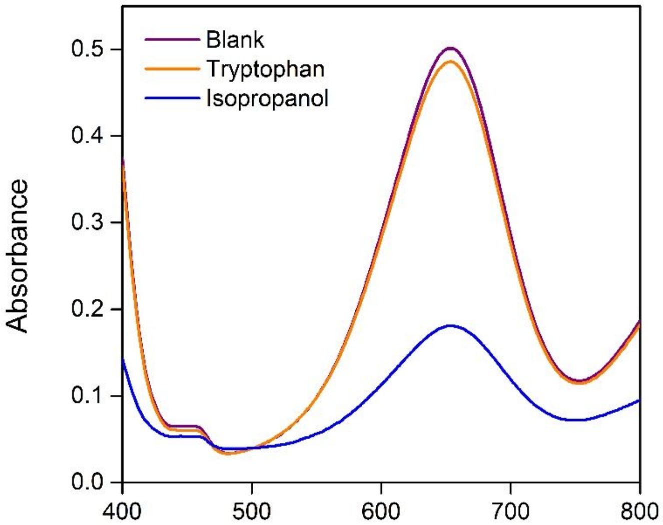

Figure S2. Absorbance spectrum of the HRP-NF@CNT-30Is-TMB-H_2O_2 system in the presence of isopropanol and tryptophan

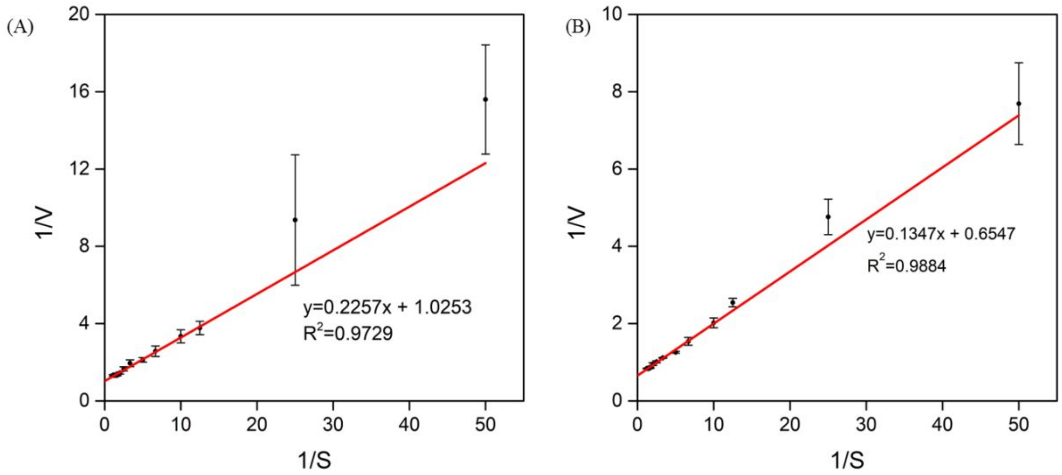

Figure S3. Lineweaver burk plot for (A) HRP-NF, (B) HRP-NF@CNT-30Is.

Table S1 List of Linear Ranges and Detection Limits for H_2O_2 of Various Materials

Materials	Linear range (μM)	LOD (μM)	Ref.
HRP enzyme	20-500	20	1
2-D PC HRP/BSA-75 hydrogel	8.8-60.6	8.8	2
HRP/poly(ANI-co-AA)	25-200	35.6	3
GO-FeTPyP/o-tolidine	20-500	72	4
Brominated graphene	500-5000	417	5
HRP-NF@CNT-30Is	20-300	2.26	This work

Table S2 List of Linear Ranges and Detection Limits for GSH of Various Materials

Materials	Linear range (mM)	LOD (μM)	Ref.
Fe-N-C SANs	0.1-0.4	78.33	6
Pt/NiCo-LDH NCs	50-500	3770	7
$Cu_{1.8}S$	0.5-10	60	8
Cu/Au NPs	0.07-0.3	13	9
HRP-NF@CNTs-30Is	0.02-0.2	11.2	This work

References

(1) Lin, Z.; Xiao, Y.; Yin, Y.; Hu, W.; Liu, W.; Yang, H. Facile synthesis of enzyme-inorganic hybrid nanoflowers and its application as a colorimetric platform for visual detection of hydrogen peroxide and phenol. *ACS Appl. Mater. Interfaces* **2014**, *6* (13), 10775-10782.

(2) Liu, R.; Cai, Z.; Zhang, Q.; Yuan, H.; Zhang, G. Colorimetric two-dimensional photonic crystal biosensors for label-free detection of hydrogen peroxide. *Sens. Actuators, B* **2022**, *354*, 131236.

(3) Hosu, O.; Lettieri, M.; Papara, N.; Ravalli, A.; Sandulescu, R.; Cristea, C.; Marrazza, G. Colorimetric multienzymatic smart sensors for hydrogen peroxide, glucose and catechol screening analysis. *Talanta* **2019**, *204*, 525-532.

(4) Socaci, C.; Pogacean, F.; Biris, A. R.; Coros, M.; Rosu, M. C.; Magerusan, L.; Pruneanu, S. Graphene oxide vs. reduced graphene oxide as carbon support in porphyrin peroxidase biomimetic nanomaterials. *Talanta* **2016**, *148,* 511-517.

(5) Singh, S.; Mitra, K.; Singh, R.; Kumari, A.; Gupta, S. K. S.; Misra, N.; Maiti, P.; Ray, B. Colorimetric detection of hydrogen peroxide and glucose using brominated graphene. *Anal. Methods* **2017,** *9* (47), 6675-6681.

(6) Lu, W.; Chen, S.; Zhang, H.; Qiu, J.; Liu, X. FeNC single atom nanozymes with dual enzyme-mimicking activities for colorimetric detection of hydrogen peroxide and glutathione. *J. Materiomics* **2022**, *8* (6), 1251-1259.

(7) Cao, X.; Yang, H.; Wei, Q.; Yang, Y.; Liu, M.; Liu, Q.; Zhang, X. Fast colorimetric sensing of H_2O_2 and glutathione based on Pt deposited on NiCo layered double hydroxide with double peroxidase-/oxidase-like activity. *Inorg. Chem. Commun.* **2021**, *123*, 108331.

(8) Zou, H.; Yang, T.; Lan, J.; Huang, C. Use of the peroxidase mimetic activity of erythrocyte-like Cu 1.8 S nanoparticles in the colorimetric determination of glutathione. *Anal. Methods* **2017**, *9* (5), 841-846.

(9) Sun, R.; Lv, R.; Zhang, Y.; Du, T.; Li, Y.; Chen, L.; Qi, Y. Colorimetric sensing of glucose and GSH using core–shell Cu/Au nanoparticles with peroxidase mimicking activity. *RSC Adv*. **2022**, *12* (34), 21875-21884.
